# Influence of Time Interval, Temperature, and Storage Condition on Fluoride Release and Recharge from Silorane-Based Restorative Materials

**DOI:** 10.3390/dj13050197

**Published:** 2025-04-30

**Authors:** Prashanthi S. Madhyastha, Dilip G. Naik, Srikant Natarajan, Rachel Sarah Vinodhini

**Affiliations:** 1 Department of Dental Materials, Manipal College of Dental Sciences, Mangalore, Manipal Academy of Higher Education, Manipal 575001, Karnataka, India; rachel.vinodhini@learner.manipal.edu; 2 Department of Periodontics, Manipal College of Dental Sciences, Mangalore, Manipal Academy of Higher Education, Manipal 575001, Karnataka, India; dilip.naik@manipal.edu; 3 Department of Oral Pathology and Microbiology, Manipal College of Dental Sciences, Mangalore, Manipal Academy of Higher Education, Manipal 575001, Karnataka, India; srikant.n@manipal.edu

**Keywords:** fluoride release, fluoride-selective electrode, methacrylate-based composites, recharge, silorane-based composites

## Abstract

**Objectives:** The fluoride-releasing properties of restorative materials are crucial for the prevention of secondary caries as these can act as fluoride reservoirs. Hence, the present study aimed to investigate, assess, and compare the impact of time, temperature, and storage conditions on the fluoride release of silorane-based composites (SBCs) and methacrylate-based composites (MBCs), and also evaluate the variation in their reuptake of fluoride (after recharge). **Methods:** SBC and MBC test samples of 10 mm × 2 mm dimensions were prepared and tested for fluoride release and recharge in distilled water and artificial saliva at temperatures of 4 °C, 37 °C, and 55 °C. The amount of fluoride released (at 1, 7, 14, and 28 days) and re-released after recharge (with 5000 ppm neutral sodium fluoride (NaF) solution for 5 min at 1, 3, and 7 days for 3 weeks) were studied with the help of a fluoride-selective ion electrode. **Results:** SBCs had a greater release of fluoride at low temperature in artificial saliva (0.07 ± 0.03) when compared to MBCs (0.04 ± 0.005). Fluoride release increased on day 7 but decreased over time (*p* < 0.05). Fluoride re-release was greater in MBCs than SBCs and it increased with time (*p* < 0.05). **Conclusion:** The amount of fluoride release and recharge depends on the time interval, temperature, and storage condition. These restorative materials can serve as fluoride reservoirs and contribute to sustained fluoride release in oral fluids, thereby preventing the initiation of secondary caries and the failure of restorations. In addition, it may assist in developing measures to improve fluoride delivery for topical applications.

## 1. Introduction

Over the years, methacrylate-based tooth-colored composite resins have been widely and effectively used in the field of restorative dentistry. These methacrylate-based composites (MBCs) are made up of a blend of silinated inorganic filler, monomeric resin matrix, polymerization initiator, and inhibitors to prevent premature polymerization during storage and color pigments [1,2]. However, they do suffer from limitations like the inevitable problems of shrinkage during polymerization (2–3% by volume) and poor clinical outcomes like decreases in marginal integrity, micro leakage, debonding, microcracks, and postoperative sensitivity. The associated complications might be solved by altering the polymerization chemistry. One such alternative is the development of innovative silorane-based composite (SBC) resin. Silorane-based composites (Filtek P 90), which comprise a ring-opening monomer, with oxirane and siloxane structural moieties, have been reported to have low polymerization shrinkage (>1%) along with improved wear resistance, optical properties, and good biocompatibility. This low shrinkage is because of the cationic ring-opening polymerization process of the cyclic epoxides. This property is desirable when developing new dental composite filling materials that will have superior properties when compared to the current resin compounds based on acrylates [2,3].

Failure of restorations due to secondary caries still remains the single most inevitable problem in restorative dentistry [4]. As a cornerstone in the prevention of dental caries, fluoride plays a crucial role in oral health promotion in both children and adults [5,6]. Fluoride-releasing restorative materials can serve as fluoride reservoirs and contribute to the low and sustained release of fluoride in the oral fluids, thereby preventing dental caries [6,7,8]. This release of fluoride ions from restorative materials could also be beneficial in enriching the enamel and dentin of the adjacent teeth, thereby enabling them to combat caries. The behavior of restorative material under conditions of varying temperature, time, and storage that simulates the conditions of the oral cavity affects the release of fluoride [9,10,11,12]. Despite their high strength, good wear resistance, and excellent esthetics, fluoride-releasing restorative materials release only a small amount of fluoride and have low fluoride recharge capability. Therefore, the anticariogenic effect of the current fluoride-releasing dental restorative materials is questionable.

There is usually a sharp decline in the release of fluoride ions from restorative materials after 3 days. The fluoride-releasing ability can be partly recharged or regenerated by the use of topical fluoride agents or fluoride-containing dentifrices. However, this recharge ability varies widely among the different classes of fluoride-releasing materials [13]. Thus, the estimation of fluoride recharge and release from restorative materials under varying conditions of temperature, time, and storage that simulates the conditions of oral cavity becomes highly important. Fluoride-releasing restorative materials have adequate mechanical and esthetic properties, but low fluoride release and recharge abilities. Hence, the anticariogenic property of the current fluoride-releasing restorative materials is uncertain.

Preston et al. 1999 [11] compared the fluoride release (FR) and recharge parameters of the esthetic restorative materials compomer, MBCs, SBCs, and glass ionomer cement (GIC). Fluoride re-release was found to be higher in GIC and in Dyract AP, and FR was higher in artificial saliva when compared with the distilled water. Further, it was found to be more significant at high temperature and at the duration of three weeks. Kelić et al. (2020) [14] determined the impact of glass ionomer resin and adhesive systems (Cention, alkasite, giomers and Filtek) on the FR and concurrent pH changes over 168 days. Fluoride release differed among the analyzed materials and depended on the utilization of coatings and dental adhesives. Further, the study observed that the pH of all the substances, time points, and coating types differed significantly. Panpisut et al. (2020) [15] evaluated the impact of the powder-to-liquid ratio (PLR) on the setting time, compressive strength, and fluoride release of SPGs (spherical glass fillers). Increasing the PLR showed decreased cumulative fluoride release. These comparative results demonstrated that increasing the PLR has an effect on the setting time, compressive strength, and fluoride release of the restorative materials and thereby demonstrated reduced results. Turjanski et al. (2023) [16] investigated the impacts of radiotherapy on the surface, chemical, and mechanical properties of restorative materials like alkasite and a glass hybrid. The study found no statistical differences and only a minor change was found in terms of discoloration. Singh, H et al. (2020) [17] compared the FR of GIC and other four restorative materials. The study used an ANOVA, post hoc test to determine the comparative analysis. Accordingly, the study also found that alkasite was much better than the other materials. The following limitations found among the existing studies clearly show that clinical case-customized implementation was not effectively performed. Likewise, the longevity of the material was generally not assessed. The effects of storage were not critically investigated by the existing studies. Challenges were associated with the low mechanical compressive strength of the material.

A drastic reduction in fluoride release is generally observed in a restorative material after three days. This feature can be partially counteracted by recharging with topical fluorides or fluoridated dentifrice [18], but the recharge differs among various classes of fluoride-releasing materials [19]. Hence, fluoride release and recharge become more important [20]. With this background, this study aimed to investigate the impact of temperature, time, and storage conditions on fluoride release followed by the recharge of restorative materials such as silorane-based composites (SBCs) and methacrylate-based composites (MBCs).

## 2. Materials and Methods

The present study compared the amounts of fluoride release and recharge capacities of SBCs and MBCs in two different storage conditions, artificial saliva and distilled water, at three temperatures, 4 °C, 37 °C, and 55 °C, over periods of 1 day, 7 days, 14 days, and 28 days. Table 1 lists the details of the composites used in the present study.

### 2.1. Test Specimen Preparation

To reduce variability, all samples were prepared by a single operator. The test samples were prepared by using Teflon molds (Hebich Technical Training Institute, Mangalore, India) of 10 mm diameter and 2 mm thickness. The mold was sandwiched between a transparent matrix strip and a glass slide on either side. The uncured restorative materials were inserted into the mold and overfilled intentionally. The excess material was expelled from the mold by applying light pressure. Each sample was light-cured through the top and bottom of the glass slides for the duration recommended by the manufacturers. The light curing unit (light-emitting diodes (LEDs)—360 mW/cm^2^; Bee Cool Plus, Confident Dental Equipment, India Pvt Ltd., Bangalore, India) had the same dimensions as the sample, which was achieved by ensuring that the sample exposure to curing light was adequate and uniform. The Hilux curing light meter (Bee Cool Plus, Confident Dental Equipment, India Pvt Ltd., Bangalore, India) was used to check the intensity of the curing light before each sample run. After they were set, the cylindrical samples were separated from the mold. All samples were stored at a relative humidity of 100% at 37 °C for 24 h.

### 2.2. Release of Fluoride

Sixty samples of MBC and SBC were prepared separately. Thirty of the samples were immersed in 20 mL of artificial saliva contained in 30 mL test tubes, and the remaining thirty samples were immersed in 30 mL test tubes containing 20 mL of distilled water. These samples were further subdivided into three groups of 10 samples each, and stored at 4 °C in the refrigerator, 55 °C in the water bath, and 37 °C in the incubator, respectively, for a period of 24 h. After 24 h, the samples were removed from the tubes, and the elutes were individually subjected to fluoride release analysis using a fluoride-sensitive electrode. (Thermo Fisher Scientific Inc., Waltham, MA, USA) The removed samples from both the solutions were washed with distilled water, and then, consequently, dried using blotting paper and then transferred to fresh jars containing 20 mL of artificial saliva or 20 mL of distilled water.

The same protocol was continued, and the fluoride ion concentration was measured on days 7, 14, and 28. The Orion™ 2109 XP Fluoride Analyzer (Thermo Fisher Scientific Inc., Waltham, MA, USA) connected to the Orion Ion Analyzer (Thermo Fisher Scientific Inc., Waltham, MA, USA) was used to measure the fluoride ion concentration. For the analysis, the elutes were buffered with a 1:1 ratio of TISAB (Total Ionic Strength Adjustment Buffer) solution and the ionic concentration of fluoride was measured. The analysis was performed thrice for each sample and the mean values were considered to obtain accurate results.

### 2.3. Recharge of Fluoride

The individual samples were recharged in a solution containing 5000 ppm of neutral NaF (sodium fluoride) for a period of five minutes and then repeated for 3 weeks. After every episode of recharge, the re-release of fluoride was estimated at the periods of one, three, and seven days.

### 2.4. Statistical Analysis

One-way ANOVA and Tukey’s post hoc test at each period were performed for the comparison of the four groups, which included SBC &/or MBC immersed in distilled water or artificial saliva. Two-way repeated-measures ANOVAs were performed to evaluate the changes over temperatures. The comparison of the data between distilled water and artificial saliva was performed using an independent t test. Data were expressed in mean and standard deviation and *p* values < 0.05 were considered statistically significant.

## 3. Results

### 3.1. For Fluoride Release

**Figure 1:**
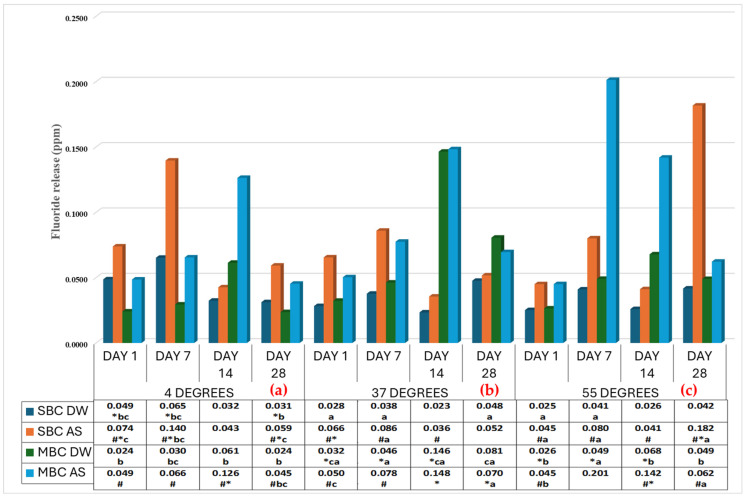
Comparison of fluoride release in all materials at all time intervals, temperatures, and storage media. #—Comparison with storage media. *—Comparison with materials. a, b, c—letters significantly differ from the temperature.

A comparison between immersion media (distilled water and artificial saliva) (Figure 1) shows that fluoride release was significantly greater (<0.001) in artificial saliva than in distilled water. Further, at lower temperatures in artificial saliva, fluoride release was greater in SBC. On the other hand, as the temperature increased, MBC showed comparatively greater fluoride release. Also, when comparing the fluoride release with regard to time period, in artificial saliva, the release was highest on day 7 for SBC, whereas day 14 showed the highest fluoride release with MBC.

A comparison between materials (SBC and MBC) irrespective of immersion media (Table 2, Figure 1) shows that at lower temperatures, SBC releases higher fluoride than MBC, but at higher temperatures, fluoride release was greater in MBC.

### 3.2. For Fluoride Recharge

The comparison between immersion media (distilled water and artificial saliva) shows that fluoride re-release was significantly higher (<0.001) in artificial saliva than in distilled water. Further, the difference in fluoride reuptake (after recharge) in artificial saliva during the weekly recharge showed that re-release progressively increased from week 1 to week 3. Also, in comparison to single days at the weekly recharge, day 1 showed higher release than day 3, while day 7 showed the lowest release.

Comparison between materials (SBC and MBC) irrespective of immersion media shows that MBC re-released higher fluoride than SBC in the first and third weeks, whereas the re-release was greater in the second week for SBC. The fluoride re-release significantly dropped from day 1 to day 3, but there was no significant difference from day 3 to day 7.

In conclusion, on comparing the fluoride release at low temperature in artificial saliva, SBC had a greater release when compared to MBC. Fluoride release increased on day 7 but decreased over time. Fluoride re-release was greater in MBC than SBC and it increased with time.

## 4. Discussion

A rise in demand for esthetics-oriented treatment from patients has revolutionized the market of esthetic materials. Materials have evolved from being not only esthetic but also having anticariogenic properties. The release of fluoride from restoratives prevents caries, enhances remineralization, and provides an anticariogenic effect. The upsurge in the concentration of fluoride ions in and around restorations is beneficial for patients with a high caries index. Fluoride reservoirs and their ability to release ions are imperative for governing the success of a topical treatment. The fluoride replaced from the milieu is re-released to the neighboring tooth structures as well [21]. The restorative material thus recharged by fluoride acts as a possible reserve for future release into the oral environment.

This study evaluated the in vitro fluoride release and the recharge potential of two restorative materials (as shown in Table 1) over an extended period of time. The fluoride release and recharge of both the composites were found to be low, and the values probably represented the amount of fluoride remaining on the surface after the recharge procedure and the wash cycles. There were several competing factors which contributed to the fluoride release pattern. The final outcome could be confounded by the cumulative changing experimental conditions in this study, such as the powder–liquid ratio, mixing, manipulation of the materials, different amount of exposed area for the specimens, the nature of the storage medium used, the weight of specimens, and the form and concentration of fluoride recharging vehicles.

Materials can leach ions from the mass that have been penetrated by water. During water penetration through diffusion, the surface layers will be more saturated than the inner mass [22]. This penetration of water is different for different materials, depending on the permeability of the materials. The diffusion of fluoride ions through a polymer matrix of composites is more demanding than glass ionomer cement. Glass ionomer cement is highly permeable and attains saturation quickly, resulting from the dissolution of the soluble fraction of material. Fluoride being taken up by the surface may explain the low fluoride release [23].

The current investigation was carried out at different temperatures to check whether a change in the surrounding temperature can influence the fluoride release from the restorative materials. The thermal circuits formed due to extensive temperature variations in the oral cavity may result in questionable longevity of the restorative materials.

The outcome of the present study suggested that the rise in temperature increases the recharge capacities and fluoride release in both MBCs and SBCs. This proposes that to effectively enhance the recharge ability of restorative materials clinically during fluoride treatment, a higher temperature is recommended. Such a treatment could be effectively used for the development of regimes for the delivery of topical fluoride.

The test medium also plays a crucial role in fluoride release. Distilled water has been used as the test medium in several studies [24,25]. It has been shown that fluoride release varies in artificial saliva compared to in distilled water. Moreover, the use of artificial saliva provides test conditions that are more comparable with the oral environment than with distilled water. Therefore, in the present study, artificial saliva was included as a test medium. The results of our study showed that greater release was observed in artificial saliva than in distilled water, suggesting the successful use of fluoride-containing restorative materials in the oral environment. This can be explained by the effect of pH of the dissolving medium on fluoride release. It has been reported that an acidic pH increases the release of fluoride ions from the restorative materials [26,27]. The artificial saliva in this study had a pH of 5.3 to 5.5, which explains the higher fluoride release in artificial saliva when compared to distilled water.

The recharge capacity of fluoride is much more significant than the release of fluoride alone. Thus, regular contact of the restorative materials with topical fluorides like acidulated phosphate fluoride (APF), stannous fluoride (SnF2), or sodium fluoride (NaF) in mouthwashes and dentifrices can potentially recharge these materials with fluoride [28]. In the current analysis, NaF was utilized as the recharging vehicle, which is also known to be a common ingredient in mouthwashes and dentifrices. The standardized concentration of fluoride is generally 1000 parts/10^6^ F. This study used 500 parts/10^6^ F for the determination of fluoride recharge effectiveness at very low concentrations. Also, the concentration used for fluoride replenishing was 0.2% NaF solution. A rise in fluoride release after exposure to 0.2% NaF solution can be attributed to fluoride being retained in pores and surfaces of the restorative materials.

**Figure 2:**
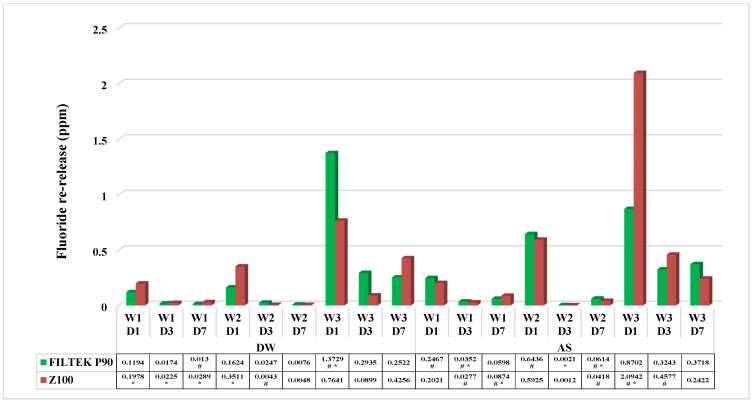
Comparison of fluoride re-release in all materials at all time intervals and storage media. #—Comparison with storage media. *—Comparison with materials.

In our study, re-release increased from week 1 to week 3, as shown in Figure 2, suggesting that the topical applications of fluoride could potentially help in the prevention of dental caries. SBC and MBC did not significantly differ, suggesting that more frequent application of fluoride is needed in composites. Several factors are likely to be involved in the process, like the permeability of the material and the form and concentration of the fluoride used [29]. If the permeability of the material is high, then the absorption and re-release of fluoride can take place to a greater extent when compared to relatively permeable material. The limitations of the current study were that the study was performed completely in an in vitro setting, which could mean that there may be variations in the fluoride release in an in vivo environment. Hence, in vivo studies are crucial to study the fluoride release profiles of restorative materials.

## 5. Conclusions

Our results suggest that fluoride-containing dental materials have long-term sustained fluoride-releasing capacity. When choosing a material for a caries-prone patient, it is important to take the release data into account rather than relying on the general class of the material. A composite needs more intermittent and more frequent application of fluoride for effective release. The use of a higher temperature during topical fluoride applications may increase the fluoride recharging and re-release ability of restorative materials, and a low oral environment temperature should be avoided during topical fluoride application. Given that fluoride release varies among materials and that most people use a fluoridated dentifrice at least once a day, using a material for recharge might represent an effective method for self-administered secondary caries prevention. Further research can be taken to investigate the fluoride-releasing and recharging ability and the performance of composites in a clinical setting and planned in vivo studies.

## Figures and Tables

**Table 1:** Materials used in the study.

Name	Type	Manufacturer	Shade	Organic Matrix	Inorganic Fillers	Filler Content [vol.%]
**Filtek P90** **[SBC]**	Silorane-based micro hybrid composite	3M/ESPE, St. Paul, MN, USA	**A2**	3,4-Epoxycyclohexylethyl cyclopolymethylsiloxane, bis-3,4-poxycyclohexylethylphenylmethylsilane	Silanized quartz, yttrium fluoride	55
**Z100** **[MBC]**	Methacrylate-based hybrid composite	3M/ESPE, St. Paul, MN, USA	**A2**	Bis-GMA and TEGDMA	Zirconium, silica	66

**Table 2:** Independent Student’s t test to compare the fluoride release between artificial saliva (AS) and distilled water (DW) at different time periods and temperatures.

			AS (Mean ± sd)	DW (Mean ± sd)	t	*p* Value
**4 Degrees**	SBC	DAY 1	0.074 ± 0.028	0.049 ± 0.017	2.425	0.028
		DAY 7	0.14 ± 0.066	0.065 ± 0.033	3.182	0.007
		DAY 14	0.043 ± 0.011	0.032 ± 0.016	1.691	0.11
		DAY 28	0.059 ± 0.015	0.031 ± 0.006	5.368	<0.001
	MBC	DAY 1	0.049 ± 0.005	0.024 ± 0.002	14.96	<0.001
		DAY 7	0.065 ± 0.004	0.029 ± 0.002	26.935	<0.001
		DAY 14	0.126 ± 0.05	0.061 ± 0.052	2.865	0.01
		DAY 28	0.045 ± 0.002	0.024 ± 0.001	27.801	<0.001
**37 Degrees**	SBC	DAY 1	0.066 ± 0.02	0.028 ± 0.002	5.837	<0.001
		DAY 7	0.086 ± 0.003	0.038 ± 0.002	41.226	<0.001
		DAY 14	0.036 ± 0.005	0.023 ± 0.001	6.903	<0.001
		DAY 28	0.052 ± 0.001	0.048 ± 0.014	0.907	0.388
	MBC	DAY 1	0.05 ± 0.003	0.032 ± 0.004	11.538	<0.001
		DAY 7	0.077 ± 0.013	0.046 ± 0.007	6.914	<0.001
		DAY 14	0.148 ± 0.032	0.146 ± 0.035	0.127	0.9
		DAY 28	0.07 ± 0.014	0.081 ± 0.049	−0.688	0.506
**55 Degrees**	SBC	DAY 1	0.045 ± 0.002	0.025 ± 0.001	35.242	<0.001
		DAY 7	0.08 ± 0.004	0.041 ± 0.004	23.386	<0.001
		DAY 14	0.041 ± 0.004	0.026 ± 0.001	11.269	<0.001
		DAY 28	0.182 ± 0.022	0.042 ± 0.013	17.58	<0.001
	MBC	DAY 1	0.045 ± 0.004	0.026 ± 0	15.683	<0.001
		DAY 7	0.201 ± 0.274	0.049 ± 0.003	1.756	0.113
		DAY 14	0.142 ± 0.01	0.068 ± 0.039	5.775	<0.001
		DAY 28	0.062 ± 0.007	0.049 ± 0.005	5.121	<0.001

## Data Availability

Data will be made available on request.

## Abbreviations

The following abbreviations are used in this manuscript:

SBC	Silorane-based composites
MBC	Methacrylate-based composites
GIC	Glass ionomer cement
TISAB	Total ionic strength adjustment buffer
NaF	Sodium fluoride
FR	Fluoride release
PLR	Powder-to-liquid ratio
LED	Light-emitting diode
AS	Artificial saliva
DW	Distilled water
APF	Acidulated phosphate fluoride
